# Non-Ocular Cancers in Parents of Patients Diagnosed with Retinoblastoma in Britain 1949 to 1987

**DOI:** 10.3390/cancers18091399

**Published:** 2026-04-28

**Authors:** Charles A. Stiller, Angela MacCarthy, Kathryn J. Bunch, Nicole L. Diggens, Gerald J. Draper, Michael F. G. Murphy

**Affiliations:** 1 National Disease Registration Service, NHS England, London SE1 8UG, UK; 2 Formerly with Childhood Cancer Research Group, Department of Paediatrics, University of Oxford, Oxford OX1 2JD, UK; 3 UConn Center on Aging, University of Connecticut Health Center, Farmington Ave., Farmington, CT 06030, USA; 4 Nuffield Department of Women’s and Reproductive Health, University of Oxford, Women’s Centre, John Radcliffe Hospital, Oxford OX3 9DU, UK

**Keywords:** child, follow-up studies, germline mutation, Great Britain, medical record linkage, mutation, neoplasms, parents, registries, retinoblastoma

## Abstract

**Background:** Mutations in the retinoblastoma gene (RB1) are associated with risks of both retinoblastoma and other cancers. Parents of children with retinoblastoma can be categorised according to their likelihood of carrying a germline RB1 mutation. Some categories of such parents may have an increased risk of cancer. **Methods:** A cohort of 1180 parents of children with retinoblastoma were categorised according to the likelihood that they carried an RB1 mutation and followed up for cancer through national records. We calculated Standardised Incidence Ratios (SIRs) for all non-ocular cancers combined and for individual diagnostic groups, with expected numbers derived from national cancer registration rates. Finally, we pooled the all-cancers results from the present study with those from an earlier study of largely the same cohort with non-overlapping follow-up. **Results:** In total, 183 non-ocular cancers were identified among the parents. Parents who themselves had retinoblastoma had a significantly higher risk of non-ocular cancer than the general population: for fathers, the SIR was 3.56 (95% confidence interval (CI) 1.84–6.22); for mothers, it was 3.25 (1.49–6.18). For the very small group of parents known to be carrying a germline RB1 mutation but not affected by retinoblastoma, there was a lower and non-significant increase in risk (SIR = 1.9). Parents categorised as either possible carriers or probable non-carriers had similar observed risks to the general population. When the all-cancers results were pooled with those from an earlier study of very largely the same cohort with non-overlapping follow-up, the estimated lifetime SIR for non-ocular cancer among mutation-carrier parents after the birth of their affected child was 4.32 (95% CI 3.06–5.93). **Conclusions:** Our results confirm that parents who themselves had retinoblastoma have an increased risk of subsequent cancers, and parents who are not mutation carriers have a risk similar to the general population.

## Simple Summary

Mutations in the retinoblastoma gene (RB1) are associated with risks of retinoblastoma and other cancers. Parents of children with retinoblastoma can be categorised according to their likelihood of carrying a germline RB1 mutation. To obtain reliable estimates of cancer risk to parents of a child with retinoblastoma after the birth of such a child, we studied a cohort of 1180 such parents. Fathers and mothers who themselves had retinoblastoma each had a threefold higher risk of non-ocular cancer than the general population. The very small group of parents known to be carrying a germline RB1 mutation but not affected by retinoblastoma had a lower, but non-significant, increase in risk. When our results were pooled with those for largely the same group of parents at younger ages, those known to be mutation carriers had an estimated fourfold higher lifetime risk of non-ocular cancer after the birth of their affected child. Parents who are not mutation carriers have a cancer risk similar to the general population.

## 1. Introduction

Retinoblastoma is an embryonal malignancy of the retina that nearly always occurs in childhood, with most cases occurring in the first five years of life, with the highest incidence being among infants under one year of age [1,2]. Age-standardised incidence among children under 15 years of age is usually in the range 3.5–6.0 per million child-years, and incidence at age 0–4 years is typically between 10 and 15 per million child-years [3,4,5,6,7,8,9]. Retinoblastoma occurs in two forms. The heritable form involves a germline mutation in one allele of the RB1 gene (which may be inherited from a parent or may be a de novo mutation), followed by an acquired mutation in the second allele. The non-heritable form involves two post-conception mutational events in one retinal cell. In industrialised countries, around 45–50% of cases are ‘heritable’ and 50–55% ‘non-heritable’ [1]. In heritable cases, there is an increased risk of second primary tumours that continues well into adulthood [10,11,12,13,14,15,16,17,18,19,20,21,22,23].

The parents of some patients with retinoblastoma will themselves have had retinoblastoma or be unaffected RB1 mutation carriers; these parents can be expected to have an increased risk of other cancers. In an earlier study of non-ocular tumours in relatives of retinoblastoma patients in Britain, parents who were known carriers of a germline mutation of RB1 had a 13-fold increased risk of cancer compared with the general population, and there were significantly raised risks for cancers of the lung and bronchus, bladder cancer, melanoma and brain tumours [24]. The objective of the present study was to determine the magnitude of the risks of cancer overall and of a wider range of cancer types among parents who had become, on average, more than 20 years older than in the previous study, subdivided according to the likelihood that they carried a germline mutation of RB1. To calculate these risks, we compared the number of cancers observed in each of these groups with those expected on the basis of general population rates.

## 2. Methods

### 2.1. Data Sources

Children with retinoblastoma diagnosed between 1949 and 1987 while resident in Britain (the ‘probands’) were identified mainly through the (UK) National Registry of Childhood Tumours (NRCT) [4]. Identifying details for parents were reported to the National Health Service Information Centre (NHSIC). (If two or more probands had the same parent, that parent was included only once in the study.) Through record linkage, we were notified of cancer registrations, embarkations from Britain, and deaths in these individuals in the period 1991–2011. The parents whose records were used for this study are largely the same as those in our previous study, in which parents (identified from the Oxford Survey of Childhood Cancers, the National Cancer Registration Scheme as it existed at the time, and the records of specialist treatment centres) were followed up to the end of 1986 for death and during the years 1971–1984 for cancer registration [24]. There is thus no overlap between the periods of surveillance for second tumours covered by the two studies, but the ages at which the parents are under surveillance in the present study are older. We included in our analyses all malignant tumours except non-melanoma skin cancers, together with non-malignant central nervous system/brain tumours, which were also routinely registered. Non-melanoma skin cancers were excluded because they were incompletely recorded by cancer registries [25]. In addition to the analyses based on this total group of tumours, we analysed results for various diagnostic groups defined by tumour site and/or morphological type. Finally, we pooled the all-cancers results from the present study with those from the earlier study [24], on the basis that this presents a better estimate of the lifetime risk to age 84 years than either of the two studies separately.

### 2.2. Mutation Category Definitions

The parents were divided into four ‘mutation categories’ on the basis of the likelihood that they were carrying an RB1 germline mutation:

Affected mutation carriers—Parents who themselves have retinoblastoma, including regressed retinoblastoma.

Unaffected mutation carriers—Parents for whom it can be inferred from their family history that they are carrying an RB1 mutation but who are not themselves known to have had retinoblastoma.

Possible mutation carriers—Two sub-categories of parents were classified as ‘possible carriers’: first, those whose only affected relatives are either a child with bilateral retinoblastoma (and, possibly, direct descendants of this child) or a child with unilateral retinoblastoma who has at least one affected direct descendant; such parents may be RB1 mutation carriers or may have a de novo germline mutation; second, unaffected parents who have more than one child carrying the mutation; for such parents, it can be inferred that each has approximately a 50% chance of being a mutation carrier.

Probable non-carriers—Two sub-categories of parents were classified as ‘probable non-carriers’: First, unaffected parents whose only affected relative is one unilaterally (or unknown laterality) affected offspring; second, unaffected parents whose partner is known to have a germline RB1 mutation.

This categorisation takes no account of the possibility of genetic mosaicism in the families; we assume that the occurrence of mosaicism would not affect our general assessment of the probability of being an RB1 mutation carrier.

### 2.3. Statistical Analysis

For the analysis of each diagnostic group, only one record was included for each parent: parents not developing a cancer in that group were included and regarded as cancer-free; for those with a record of more than one relevant cancer, only the first was included. Observed numbers of cancers were compared with numbers that would be expected on the basis of cancer registration rates for England and Wales, 1991–2006 (for the years 2007–2011, we used data for 2006). The at-risk period was defined in terms of dates of entry to, and exit from, the study. ‘Entry date’ was defined as the start of surveillance through the NHSIC (1 January 1991). (All of the probands were born before this date; thus, all parents were entered into surveillance after the birth of the child.) ‘Exit date’ was the earliest of: the end of NHSIC surveillance for this study (1 May 2011)**,** the occurrence of a cancer in the diagnostic group being analysed, embarkation from Britain, or death. Standardised Incidence Ratios (SIRs), defined here as the ratio of the observed number of cancers to the number expected, and 95% confidence intervals (CI) were calculated using the Stata Statistical Software (Release 11; StataCorp LP, College Station, TX, USA). Two-sided significance tests of the hypothesis that the specified groups of parents have the same cancer rates as the general population are calculated assuming that the observed numbers of cases follow Poisson distributions with means calculated from cancer registration rates. Our earlier study was based on substantially the same cohort with non-overlapping follow-up, and expected numbers of cancers were derived from age-specific cancer registration rates for England and Wales in the same way as for the present study [24]. For the combined analysis of this study with the earlier one, we summed the numbers of observed and expected cancers across the two studies and calculated the SIRs by the same method as described above. The mutation categories of affected and unaffected carriers were combined for these calculations since the earlier study did not assess risk for those two categories separately.

## 3. Results

### 3.1. Overview

Table 1 shows the number of parents and observed person-years at risk in the RB1 mutation category. The mean observed at-risk period was 16.9 years; this varied between 13.9 and 17.4 years for the four mutation categories.

**Table 1:** Parents included in the study.

Parental RB1 Mutation Category *	Fathers		Mothers		Parents	
	N	Person-Years at Risk	N	Person-Years at Risk	N	Person-Years at Risk
Affected mutation carriers	32	474.52	31	474.75	63	949.27
Unaffected mutation carriers	6	64.73	8	129.38	14	194.11
Possible mutation carriers	186	3163.58	217	3867.60	403	7031.18
Probable non-carriers	326	5408.09	374	6402.93	700	11,811.02
Total	550	9110.92	630	10,874.66	1180	19,985.58

* For definitions of these groups, see Section 2; person-years at risk for each decade of parental age are shown in Table 2. The expected number of cancers in each decade is calculated using these person-years and the cancer registration rates for each decade.

**Table 2:** Person-years at risk by decade of parental age.

Person-Years at Risk by Decade of Parental Age	Fathers	Mothers	Parents
15–24 years	-	7.18	7.18
25–34 years	106.03	209.53	315.56
35–44 years	861.51	1230.10	2091.61
45–54 years	2318.21	2899.02	5217.23
55–64 years	3099.95	3361.74	6461.69
65–74 years	2100.52	2283.53	4384.05
75–84 years	624.70	883.57	1508.27
Total	9110.92	10,874.66	19,985.58

The total number of observed and expected non-ocular cancers in the parents, together with SIRs, is shown in Table 3. SIRs for diagnostic groups for which there is at least one observed cancer among affected parents or unaffected parents who are RB1 mutation carriers are in Table 4. Results for fathers and mothers are combined here. RB1 mutation carriers account for all except one of the groups, for which a statistically significant excess was found.

### 3.2. Affected Mutation Carriers

Most of the statistically significant findings were found in parents who themselves had retinoblastoma. For these 63 parents, the SIR for all cancer types combined was significantly raised, and the risk was similar for fathers, SIR = 3.56, *p* < 0.01, and mothers, SIR = 3.25, *p* < 0.01 (Table 3).

We estimated SIRs for the diagnostic groups for which there is at least one observed cancer among affected parents (Table 4). Only one such parent developed osteosarcoma, but the SIR = 662.25 (*p* < 0.01) was notable. Four parents developed soft tissue sarcomas, SIR = 64.25, *p* < 0.001; the SIRs were significantly raised, *p* < 0.01, in both fathers and mothers. Three of the four soft-tissue sarcomas were leiomyosarcomas; SIR = 168.16, *p* < 0.01. Six parents developed bladder tumours (all carcinomas), SIR = 24.62, *p* < 0.001; the SIRs for fathers (20.4 based on four cases) and mothers (42.4, two cases) were both significantly increased (*p* < 0.01). Two parents (both fathers) developed cancer of the stomach (one adenocarcinoma, the other being one of the leiomyosarcomas already referred to); SIR = 10.47 for both parent groups combined, *p* < 0.05. Four mothers developed breast cancer, SIR = 3.79, *p* < 0.05. The risks for cancers of the gastrointestinal tract overall, colon, trachea/bronchus/lung, ovary, testis and prostate were increased but not significantly.

### 3.3. Unaffected Mutation Carriers

Among these 14 parents, a total of three cancers were observed, a non-significant increase (Table 3). Of these, two were in the trachea/bronchus/lung group, a significant excess, SIR 8.59, *p* < 0.05 (Table 4).

### 3.4. Possible Mutation Carriers/Probable Non-Carriers

The only notable finding amongst the 1103 parents in these categories was the SIR of 3.53, *p* < 0.01, for leukaemia among probable non-carriers (Table 4). Of the 11 leukaemias observed amongst the parents overall, nine (three acute myeloid, two chronic myeloid and four chronic lymphocytic) occurred among such parents.

### 3.5. Results from Pooling the Two Studies

The SIR for all cancers combined among carriers estimated from the pooled data of both studies was 4.32, substantially lower than the SIR of 13.0 from the 1989 study but somewhat higher than the SIR of 3.11 from the present study alone (Table 5). As in each of the two studies separately, there was little indication that the overall risks of cancer among possible mutation carriers and probable non-carriers differ from those in the general population.

## 4. Discussion

The risk of subsequent tumours in patients who themselves had heritable retinoblastoma is well established; the risk for such patients diagnosed in Britain from 1951 to 2004 was 13 times that of the general population (SIR 13.7) [18]. In our previous study of almost the same group of parents as that reported here—but covering an earlier period of follow-up to younger ages at diagnosis and, hence, independent of the present paper’s results—the SIR for parents who had a mutation in the RB1 gene was very similar (SIR 13.0), even though the spectrum of cancers occurring among these parents in adulthood differed markedly from that of subsequent tumours occurring from childhood onwards among survivors of heritable retinoblastoma [24]. Notably, while osteosarcoma is one of the most frequent subsequent tumours in young survivors of heritable retinoblastoma, the rarity of osteosarcoma among parents of children with retinoblastoma is unsurprising given that most of the person-years of risk for parents are outside the age range at which osteosarcoma is most frequent.

In the present paper, most of the ‘at-risk’ periods during which parents were under-surveillance related to age groups older than those covered by our earlier studies [18,24]. The cancer rate for individuals with heritable retinoblastoma continues to be higher than that for the general population throughout life, although the SIR decreases with increasing age because the cancer types with the highest SIRs are less frequent at older ages: the age-specific rate associated with the RB1 mutation increases proportionately less than the population rate as age increases. Statistically significant findings for all cancers combined occurred only in the group of parents themselves diagnosed with retinoblastoma. For both fathers and mothers, the risk was approximately three times that of the general population. The smaller, non-significant excess found in the unaffected carrier parents (three cancers in 14 parents) seems consistent with our previous study, where we found no cancers in a younger group of such parents [24]. Little weight can be attached to the excess of cancers of the trachea, bronchus and lung among the unaffected mutation carriers since such a finding might easily arise by chance when a large number of diagnostic groups are being examined.

In other studies of the risk of cancer in parents of children with retinoblastoma, numbers are usually small, and parents are classified in varying ways. However, limited comparisons may be made with their findings.

All cancers: Olsen et al. studied 318 unaffected parents of retinoblastoma patients in Denmark [26]. Parents were divided into two groups—those where the proband was classified as ‘genetic’ (probands who were bilateral where ‘a distinct multifocality was observed in a unilateral tumor’, or where there was a family history) and ‘nongenetic’. Observed and expected numbers of cancers were calculated for these two categories. Parents who had had retinoblastoma themselves were excluded (i.e., they excluded the group where we found most of the non-ocular cancers). There was a deficit of cancer among these unaffected parents of ‘genetic’ probands (observed = 3; expected = 6.36). Such parents could include any of our other three categories. They also found no important excess of cancer in parents of ‘nongenetic’ probands: observed = 19; expected = 17.32.

DerKinderen et al. found no overall increase in the unaffected parents of 103 cases of ‘hereditary’ retinoblastoma in the Netherlands [27].

Leukaemia: The excess of leukaemia in parents in our study who were assumed not to have a germline RB1 mutation has no obvious explanation and, though statistically highly significant, may be due to chance. The SIR of 3.70 for leukaemia among fathers was statistically significant; the SIR of 3.41 for mothers was similar but not statistically significant. Neither Olsen et al. nor DerKinderen et al. reported cases of leukaemia in the parents in their studies [26,27].

Multiple myeloma: Olsen et al. reported two cases of multiple myeloma in fathers of ‘non-genetic’ probands [26]. We observed only one case of multiple myeloma in the parents in our study, compared with an expected number of 2.43.

Sarcomas: In a recent case–control study comparing the frequency of germline pathologic variants between patients with any type of sarcoma and cancer-free controls, a pathologic variant of RB1 was found in 0.6% of sarcoma patients and 0.04% of controls (OR = 14.67, 95% CI = 0.76 to 861.86) [28]. Olsen et al. reported one case of osteosarcoma in the mother of a ‘non-genetic’ proband [26]. DerKinderen et al. reported two cases of retroperitoneal rhabdomyosarcoma in affected parents (one father and one mother), an extremely rare diagnosis among adults [9]. In contrast to the present study, no cases of leiomyosarcoma were reported in the Danish or Dutch studies [26,27], although there is an especially high risk of leiomyosarcoma among survivors of heritable retinoblastoma [18].

Melanoma: Olsen et al. reported four melanomas in parents of children with ‘non-genetic’ retinoblastoma, SIR 9.1 (95% CI 2.9 to 21.9) [26]. We observed three melanomas in parents assumed not to have a germline RB1 mutation, but the risk was not significantly raised, SIR 1.06. DerKinderen et al. observed no melanomas [27]. There is a well-documented excess of melanoma among survivors of heritable retinoblastoma [15,18,23,29,30], which is less pronounced but still present among survivors who had not received radiotherapy [15,30]. Therefore, the absence of melanoma in parents who were mutation carriers in the present study may be surprising given that 82% (63/77) of carriers had themselves had retinoblastoma. However, while population incidence of melanoma increases with age, the SIR of melanoma following retinoblastoma decreases with attained age [15,23,30]. It should also be noted that our 1989 study contained—in addition to the two cases of melanoma in parents who were mutation carriers that were included in the risk calculations—one further case of melanoma in a mutation-carrier parent that was excluded from those calculations because it was diagnosed after the end of the period for follow-up by cancer registration [24].

Cancers of the pancreas: DerKinderen et al. reported that unaffected fathers of children with ‘hereditary’ retinoblastoma had a significantly increased risk (SIR = 8.3) of pancreatic cancer; they commented that ‘There is some evidence that the occurrence of cancer of the pancreas is related to hereditary melanoma … a tumor which is also encountered as non-ocular cancer in retinoblastoma survivors’ [27]. Olsen et al. reported a cancer of the pancreas in an unaffected father [26]. We observed no cases of cancer of the pancreas in our study cohort.

There have been very few studies of subsequent cancer risk in the parents of children with retinoblastoma. All such studies made use of population-based data on cancer occurrence in the general population of their own countries. The present study cohort was much larger than those in the studies from Denmark and the Netherlands. Most parents in the present study were followed up to a much greater age than in our earlier British study. The principal limitation of the present study is the absence of molecular confirmation of RB1 mutation in the parents, with carrier status derived solely from family history. Consequently, although a substantial proportion of cases of bilateral retinoblastoma are due to a new mutation in RB1, the number of parents who were known to be unaffected mutation carriers was very small. The confidence intervals for their relative risk were correspondingly wide, and therefore, the question of whether their cancer risk is less than that in affected carriers or is indeed similar to that in the general population remains unanswered.

## 5. Conclusions

It is clear, as with studies of retinoblastoma survivors, that carriers of the RB1 mutation have an increased risk of developing other cancers. In general, the Danish and Dutch studies agree with our results in finding no overall excess among parents if those who themselves have retinoblastoma are omitted [26,27]; this does not exclude the possibility that unaffected carriers of an RB1 mutation have an increased risk, as in our study, the number of such parents was small, and in the other studies, this group was not separately analysed [26,27]. However, survivors of heritable retinoblastoma who received radiotherapy have a higher risk of subsequent cancers than those who did not [19,20,21]. Mutation carriers who do not develop retinoblastoma may well have a lower risk of developing other tumours than do the affected carriers, perhaps because they are less likely to have received radiotherapy.

Agreement with other studies suggests that our results are not due to bias. Record linkage studies avoid some of the sources of bias to which cohort studies are liable. The most obvious problems are the likely incompleteness of cancer registration and failures in the record linkage process. But it seems reasonable to assume that the first would equally affect the four study groups and the baseline rates and so would not bias the SIRs or comparisons between them. Failure in the record linkage process would lead to an underestimate of the rates but would not affect our main conclusions. The pooled data of our two studies give an estimated lifetime SIR of 4.3 for RB1 mutation carriers. Unsurprisingly, this is considerably lower than the SIR of 13 from the 1989 study, in which a much larger proportion of the follow-up covered the younger age range in which the risk of subsequent cancers among survivors of heritable retinoblastoma is at its highest [24].

## Figures and Tables

**Table 3:** Observed and expected number of all non-ocular cancers combined among parents of retinoblastoma patients.

Parental RB1 Mutation Category *	N	Person-Years at Risk	Observed Tumours (O)	Expected Tumours (E)	SIR † (O/E)	95% CI ‡	*p*-Value
Fathers							
Affected mutation carriers	32	474.5	12	3.37	3.56	1.84–6.22	<0.01
Unaffected mutation carriers	6	64.7	2	0.53	3.77	0.46–13.63	0.17
Possible mutation carriers	186	3163.6	33	29.52	1.12	0.77–1.57	0.53
Probable non-carriers	326	5408.1	53	57.70	0.92	0.69–1.20	0.53
Mothers							
Affected mutation carriers	31	474.8	9	2.77	3.25	1.49–6.18	<0.01
Unaffected mutation carriers	8	129.4	1	1.04	0.96	0.02–5.33	0.97
Possible mutation carriers	217	3867.6	25	30.24	0.83	0.54–1.22	0.32
Probable non-carriers	374	6402.9	48	52.71	0.91	0.67–1.21	0.51
Parents							
Affected mutation carriers	63	949.3	21	6.14	3.42	2.12–5.23	<0.001
Unaffected mutation carriers	14	194.1	3	1.57	1.90	0.39–5.57	0.34
Possible mutation carriers	403	7031.2	58	59.76	0.97	0.74–1.26	0.82
Probable non-carriers	700	11,811.0	101	110.40	0.91	0.75–1.11	0.36

* For definitions of these groups, see Section 2; † Standardised Incidence Ratio; ‡ 95% confidence interval for ratio O/E.

**Table 4:** Observed and expected numbers of tumours occurring among parents in each mutation category.

Mutation Categories * and Tumour Groups	Observed Tumours (O)	Expected Tumours (E)	SIR † (O/E)	95% CI ‡	*p*-Value
Affected carriers					
Osteosarcoma	1	0.0015	662.25	16.77–3689.83	<0.01
Soft tissue sarcoma	4	0.06	64.25	17.51–164.50	<0.001
Leiomyosarcoma	3	0.02	168.16	34.68–491.44	<0.01
Bladder	6	0.24	24.62	9.04–53.60	<0.001
Trachea/bronchus/lung	3	0.99	3.03	0.62–8.85	0.14
Female breast	4	1.06	3.79	1.03–9.70	<0.05
Testis	1	0.03	36.21	0.92–201.73	0.10
Gastrointestinal tract	3	1.45	2.06	0.43–6.03	0.29
Stomach	2	0.19	10.47	1.27–37.81	<0.05
Colon	1	0.47	2.15	0.05–11.98	0.53
Ovary	1	0.17	6.03	0.15–33.58	0.24
Prostate	1	0.80	1.25	0.03–6.99	0.83
Unaffected carriers					
Bladder	1	0.05	19.38	0.49–107.98	0.12
Trachea/bronchus/lung	2	0.23	8.59	1.04–31.02	<0.05
Possible carriers					
Soft tissue sarcoma	1	0.51	1.95	0.05–10.84	0.57
Liposarcoma	1	0.07	14.20	0.36–79.10	0.14
Leukaemia	2	1.39	1.43	0.17–5.18	0.64
Central nervous system/brain	1	1.54	0.65	0.02–3.62	0.63
Trachea/bronchus/lung	12	9.55	1.26	0.65–2.20	0.46
Female breast	8	10.38	0.77	0.33–1.52	0.43
Uterus (corpus or unspecified)	1	1.68	0.60	0.02–3.33	0.56
Gastrointestinal tract	10	14.09	0.71	0.34–1.31	0.24
Stomach	2	1.84	1.09	0.13–3.94	0.91
Colon	7	4.75	1.47	0.59–3.04	0.35
Cervix	1	0.56	1.77	0.05–9.89	0.62
Ovary	2	1.64	1.22	0.15–4.40	0.79
Multiple myeloma	1	0.82	1.23	0.03–6.83	0.85
Prostate	10	6.91	1.45	0.69–2.66	0.29
Probable non-carriers					
Melanoma of the skin	3	2.84	1.06	0.22–3.08	0.93
Leukaemia	9	2.55	3.53	1.61–6.70	<0.01
Central nervous system/brain	1	2.71	0.37	0.01–2.05	0.20
Bladder	2	4.75	0.42	0.05–1.52	0.13
Trachea/bronchus/lung	15	18.01	0.83	0.47–1.37	0.46
Female breast	19	17.54	1.08	0.65–1.69	0.73
Uterus (corpus or unspecified)	2	2.91	0.69	0.08–2.48	0.56
Gastrointestinal tract	17	26.43	0.64	0.38–1.03	0.07
Stomach	3	3.55	0.84	0.17–2.47	0.76
Colon	8	8.86	0.90	0.39–1.78	0.77
Cervix	2	0.93	2.15	0.26–7.77	0.37
Ovary	3	2.86	1.05	0.22–3.06	0.94
Prostate	16	13.29	1.20	0.69–1.96	0.48

* For definitions of these groups, see Section 2; † Standardised Incidence Ratio; ‡ 95% confidence interval for ratio O/E.

**Table 5:** Observed and expected numbers of all cancers combined occurring among parents in each mutation category across the 1989 study [24] and the present study.

Study	Mutation Category *	Observed Cancers (O)	Expected Cancers (E)	SIR † (O/E)	95% CI ‡
1989	Carriers	14	1.08	13.0	7.1–21.7
	Possible carriers	17	11.4	1.5	0.9–2.4
	Probable non-carriers	24	22.9	1.0	0.7–1.6
2026	Carriers	24	7.71	3.11	1.99–4.63
	Possible carriers	58	59.76	0.97	0.74–1.26
	Probable non-carriers	101	110.4	0.91	0.75–1.11
1989 and 2026 combined	Carriers	38	8.79	4.32	3.06–5.93
	Possible carriers	75	71.16	1.05	0.83–1.32
	Probable non-carriers	125	133.3	0.94	0.78–1.12

* For definitions of these groups, see Section 2; † Standardised Incidence Ratio; ‡ 95% confidence interval for ratio O/E.

## Data Availability

The individual-level data used for this study are not publicly available because of the necessity to protect the confidentiality of patients’ information. The Childhood Cancer Research Group closed in 2014. Long-term archiving of the NRCT database is currently under negotiation.
